# Body size perception, knowledge about obesity and factors associated with lifestyle change among patients, health care professionals and public health experts

**DOI:** 10.1186/s12875-021-01383-2

**Published:** 2021-02-15

**Authors:** Virginija Zelenytė, Leonas Valius, Auksė Domeikienė, Rita Gudaitytė, Žilvinas Endzinas, Linas Šumskas, Almantas Maleckas

**Affiliations:** 1 Department of Preventive Medicine, Medical Academy, Lithuanian University of Health Sciences, A. Mickevičiaus st. 9, LT-44307 Kaunas, Lithuania; 2 Department of Family Medicine, Medical Academy, Lithuanian University of Health Sciences, A. Mickevičiaus st. 9, LT-44307 Kaunas, Lithuania; 3 Department of Surgery, Medical Academy, Lithuanian University of Health Sciences, A. Mickevičiaus st. 9, LT-44307 Kaunas, Lithuania; 4 Institute of Health Research, Medical Academy, Lithuanian University of Health Sciences, Kaunas, Lithuania; 5 Department of Gastrosurgical Research and Education, Sahlgrenska Academy, University of Gothenburg, Bla Straket 5, SE-41345 Gothenburg, Sweden

**Keywords:** Attitudes, Obesity, Body image, Body size perception, Patients, General practitioners, Nurses, Public health experts

## Abstract

**Background:**

The attitudes towards obesity may have an important role on healthier behavior. The goal of the present study was to explore the attitudes towards obesity and to investigate how these attitudes were associated with lifestyle-changing behavior among the patients attending primary care centers, health care professionals and public health experts.

**Methods:**

This cross-sectional survey study was performed in 10 primary care offices in different regions in Lithuania and in 2 public health institutions. Nine hundred thirty-four patients, 97 nurses, 65 physicians and 30 public health experts have filled the questionnaire about attitudes towards obesity and presented data about lifestyle-changing activities during last 12 months. The attitudes were compared between different respondent groups and factors associated with healthier behaviors were analyzed among overweight/obese individuals in our study population.

**Results:**

Participants failed to visually recognize correct figure corresponding to male and female with obesity. Majority of respondents’ perceived obesity as a risk factor for heart diseases and diabetes but had less knowledge about other diseases associated with weight. About one third of respondents changed their lifestyle during last 12 months. Overweight individuals with age < 45 years (OR 1.64, 1.06–2.55; *p* = 0.025) were more likely and those who overestimated current weight (OR 0.44, 0.20–0.96; *p* = 0.036) less likely to change their lifestyle. Disappointment with their current weight (OR 2.57, 1.36–4.84; *p* = 0.003) was associated with healthier behavior among participants with obesity.

**Conclusion:**

Participants had similar body size perception and knowledge about obesity. Younger age had significant association with lifestyle changing behavior among overweight individuals and disappointment with current weight among obese participants.

## Background

The prevalence of obesity is increasing worldwide and has nearly tripled since 1975 [1]. Obesity is the well-known risk factor for many diseases including type 2 diabetes mellitus (T2DM), hypertension, atherosclerotic diseases and some types of cancer. It has detrimental effect on life expectancy and is associated with increased medical costs, which has a direct negative impact on public health systems [2, 3]. Despite all this, it is a condition with substantial unmet medical needs due to lack of agreement among various guidelines, shortfalls in obesity-related clinical training and complexities in reimbursement of services [4].

Representative quality of care survey in US-ambulatory care settings revealed that nearly 50% of Patients Record Forms lacked complete weight and height data and obesity diagnosis was not reported in 70% of patients with BMI ≥30.0 [5]. The frequency of counselling for diet, exercise or weight reduction increased from 37% among patients with BMI ≥30.0 to 55% among those with reported obesity diagnosis [5]. However, underreported diagnosis or counselling activities in survey studies may not reflect real situation about obesity care. Patient-doctor relationship is core in such care and is rather complex. Evaluation of it should include also process measures such as shared decision making on when the patient is ready to tackle obesity based on patient’s weight loss history and/or patient’s presence in particular stage of change. In weight control as well as in some other problem behaviors before acting patients go through precontemplation and contemplation stages [6]. It was shown that increase in pros drives patients from precontemplation to contemplation stage and decrease in cons – from contemplation stage to action [6]. In order to increase pros of changing behaviour among patients with obesity the GPs should address patients’ perception of obesity as a major health issue [7] and estimate real weight status as there is an observed tendency towards visual biases in judging the body weight [8]. However, the major concern is the fact, that primary care physicians spent a great portion of the visit time on technical tasks and more time on discussing exercise than educating patients with obesity about their health [9].

In this context communication between the patient and the doctor is crucial. Health professionals such as primary care doctors and nurses may give adequate information to the patient taking into account patient’s stage of change. However, the nurses and doctors themselves may fail to recognize obesity because they compare their patient body dimensions to newly accepted reference size, which is culturally appropriate and became common standard in the community [10]. Furthermore, on the national scale similar problems may be encountered among public health experts (PHE) and may have an effect on the future strategies about obesity prevention and treatment.

The goal of the present study was to explore the attitudes towards obesity among the patients attending primary care centers, as well as to get better insight into attitudes of health care professionals and PHE. Also, we investigated how these perceptions, attitudes were associated with lifestyle-changing behavior among individuals with overweight and obesity in our study population.

## Methods

This study was performed as a cross-sectional survey on attitudes towards obesity and related health risks with comparison between patients, nurses, physicians and PHE.

At the moment of the study initiation the Department of Family Medicine, Lithuanian University of Health Sciences had teaching centers in 15 primary care offices and 11 centers were randomly selected to participate in this survey. Finally, the data were collected from 10 primary care centers in different regions in Lithuania (Kaunas 3, Šiauliai 1, Panevėžys 1, Kaišiadorys 1, Kėdainiai 1, Biržai 1, Skuodas 1, Pagėgiai 1). The patients were eligible, if they were 18 years or older, attended primary care office for chronic or acute condition. The healthcare professionals were eligible if they were in practice for more than 2 years and were involved in the patient care. Researchers and professionals who monitor and diagnose the health concerns as well as promote healthy practices and behaviors on the population level were defined as PHE and were recruited from public health related departments of Lithuanian University of Health Sciences as well as from Lithuanian Hygiene Institute in Vilnius. Interviewing survey took place between November 2018 and November 2019. All respondents provided informed consent before participating in the survey. Bioethical approval (No. BE-2-76, 2018.10.08) was obtained from Kaunas Regional Bioethical Committee.

### Questionnaire

In this study we followed the methodology which was developed and previously used by Public Health England experts. This questionnaire was applied to measure public attitudes to obesity in UK [11]. The official permission was obtained from Public Health England to use the questionnaire for survey in Lithuania. The translation of questionnaire into Lithuanian language was performed by two translators. One was aware of the concepts the questionnaire intends to measure and the other was naive translator. Inconsistencies between the translations were discussed and resolved, and questionnaire was back translated by 2 independent translators. After backward translation the same process with review of disparities of translations was performed and final version of translated questionnaire was produced and piloted.

The questionnaire survey form covered 40 questions on perceptions and understanding of obesity, responsibility and possible solutions, as well as social stigma issues. All participants also were asked to report their weight and height. The main reason for choosing self-reported data was the fact that we were unable to provide uniform measurements in the different settings and between different participants’ groups. The BMI was calculated by dividing weight by height in square meters. In the current article we have provided research analysis only for some questionnaire items: a) perceptions of others weight; b) perception of own body weight; c) satisfaction on the own body weight; and d) understanding of causes and health risks associated with obesity.

In order to evaluate ability to recognize obesity in others’ the participants were given two sets of pictures with 8 figures presented in order of increasing body mass index (BMI). One set of male figures and the other of females. The participants were asked at what point the picture shows a male/female figure, which is called obese. In both cases correct answer was a figure with BMI just over 32. The participants were also given the same pictures of females and males (as appropriate) and were asked, which figure best represents their own body size. The answers about individual’s body size were compared with self-reported BMI.

Further, the respondents were asked what they think about their own current weight: is he/she very underweight, underweight, about the right weight, a bit overweight or very overweight. When analyzing own-weight perceptions we compared it with actual self-reported BMI and separately presented results for normal weight, overweight and participants with obesity. The other question was asked on satisfaction by the own body weight. It was asked how happy the respondents are with their current weight: very happy, happy, neutral, unhappy or very happy. The answers were grouped according to BMI of respondents – normal weight, overweight and obesity.

The list of ten possible diseases (heart disease, high blood pressure, diabetes, stroke, arthritis, depression, some cancers, liver disease, asthma and zoster) was presented and the respondents were asked, which conditions individuals with obesity are more likely to get. The question about zoster was included to show for the respondents that not all presented diseases are associated with obesity. The knowledge about causes of obesity was explored asking, if respondents strongly agree, agree, neither agree nor disagree, disagree or strongly disagree with the statements, that people become obese because they eat too much, exercise too little, have low metabolism or inherit it.

Finally, the respondents in all groups were asked, if during last 12 months they have changed their lifestyle behavior. In case, if individuals have changed their lifestyle they were asked, if those changes were associated with healthier diet, increased physical activity or both.

### Statistical analysis

We intended to include 1200 patients, 100 nurses, 70 GPs and 30 public health experts based on statistical power, cost and feasibility of recruitment. Statistical analysis was performed using SPSS (IBM, version 25.0). Only data from those who completed the survey were included in the analyses. Data were presented as means (standard deviations), medians, and frequencies, and compared by chi square, ANOVA tests among respondent groups, when appropriate. *P* < 0.01, using 2-tailed tests was considered statistically significant. Forward conditional binary logistic regression analysis was performed to identify independent factors associated with lifestyle changes during last 12 month. Factors were included into multivariate regression analysis, if during univariate analysis *p* value was < 0.05.

## Results

**Fig. 1:**
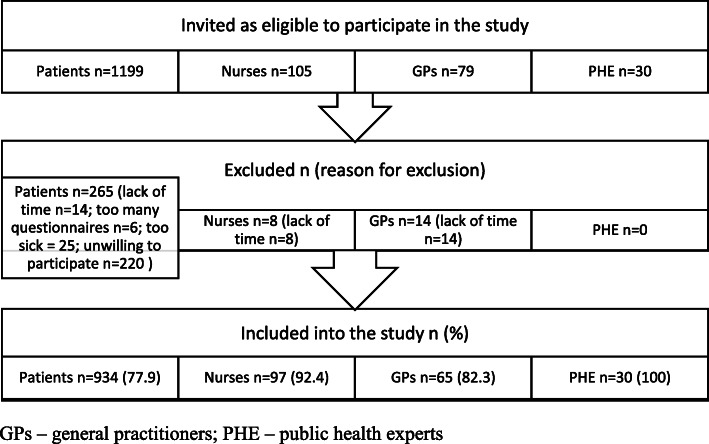
Study flow diagram

In total, 1199 patients, 105 nurses, 79 GPs and 30 PHE were invited as eligible to participate in the study. Finally, 934 patients (77.9% of all invited), 97 nurses (92.4%), 65 general practitioners (82.3%) and 30 (100%) PHE filled in questionnaires (Fig. 1). Characteristics of patients, nurses, physicians and PHE are presented in Table 1. Patients and PHE were significantly younger (41.9 and 39.9 years, respectively) in comparison with GPs and nurses (47.1 and 47.1, respectively). Fewer male participants were involved among all groups of respondents. On average, respondents had BMI 26.4 (5.8). BMI was highest in the group of patients 26.5 (6.0) and lowest – in the group of public health professionals 24.4 (4.7). There was no significant difference for average self-reported BMI and self-reported BMI category. Sixty percent of the patients had higher education. Significantly higher monthly incomes were observed in GPs and PHE groups.

**Table 1:** Sociodemographic characteristics of the study population and lifestyle changes during last 12 months

	Patients	Nurses	GPs	Public health experts	*P* value
N	934	97	65	30	
Age, years (SD)	41.9 (13.1)	47.1 (10.9)	47.1 (12.5)	39.9 (13.0)	0.001
Age					< 0.001
- 18–34	284	12	14	14	
- 35–44	206	23	12	3	
- 45–54	259	31	13	6	
- 55–64	153	29	19	4	
- 65–74	17	0	4	1	
- > 75	1	0	0	0	
Gender					0.001
Male	253	1	9	9	
Female	677	96	56	21	
Self reported BMI, average (SD)	26.5 (6.0)	26.3 (4.9)	26.1 (4.6)	24.4 (4.7)	0.282
Self reported BMI category n (%)					0.348
< 24.9	401 (44.8)	53 (54.6)	26 (40.6)	12 (46.2)	
25–29.9	297 (33.1)	25 (25.8)	25 (39.1)	11 (42.3)	
> 30	198 (22.1)	19 (19.6)	13 (20.3)	3 (11.5)	
Education					< 0.001
Secondary school or lower	369 (40.0)	0	0	0	
College or university	554 (60.0)	97 (100)	65 (100)	30 (100)	
Monthly incomes					< 0.001
< 500 euros	433 (47.5)	48 (49.5)	6 (9.2)	4 (13.3)	
≥ 500 euros	479 (52.5)	49 (50.5)	56 (91.8)	26 (86.7)	
Lifestyle changes during last 12 month n (%)					
Healthier diet	94 (10.2)	6 (6.3)	4 (6.2)	2 (6.7)	0.867
Increased physical activity	27 (2.9)	4 (4.2)	2 (3.1)	2 (6.7)	
Both	218 (23.7)	21 (22.1)	16 (24.6)	7 (23.3)	
No changes	580 (63.2)	64 (67.4)	43 (66.1)	19 (61.3)	
Changed lifestyle by weight category yes/no					
Normal weight	118/282	16/36	8/18	4/8	0.989
Overweight	115/179	6/18	7/18	5/6	0.364
Obese	97/100	9/10	7/6	2/1	0.921

### Perceptions of others weight

Only 8.1 and 12.3% of all respondents correctly identified the exact male and female figure, respectively, which was the first on the picture to present individual with obesity (Table 2). The nurses (17.5%) and doctors (15.4%) were more precise than patients (6.9%) and PHE (0%) to recognize the figure of male with obesity. The correct response rate for the figure of the female with obesity was similar between the groups. The figure with BMI around 42 was median response in all participants groups for males and in patients’ and nurses’ groups for females. Only GPs and PHE were slightly more precise in defining obesity in females with BMI around 36 as median response (Table 2).

**Table 2:** Perception of others weight: distribution of answers of respondents who identified obesity by the pictures of figures of males and females, n (%)

Group of respondents	BMI and body images on the picture^**a**^, n (%)				
	≤26^**a**^	28^**a**^	32^**a**^	36^**a**^	≥ 42^**a**^
**Male body images**					
Patients	20 (2.1)	19 (2.0)	64 (6.9)	106 (11.3)	725 (77.6)
Nurses	3 (3.1)	6 (6.2)	17 (17.5)	16 (16.5)	55 (56.7)
Doctors (GPs)	1 (1.5)	4 (6.2)	10 (15.4)	13 (20.0)	37 (56.9)
Experts	0	0	0	6 (20)	24 (80)
***Total***	*24 (2.1)*	*29 (2.6)*	*91 (8.1)*	*141 (12.5)*	*841 (74.7)*
**Female body images**					
Patients	40 (4.3)	65 (7.0)	112 (12.0)	144 (15.4)	573 (61.3)
Nurses	9 (9.3)	11 (11.3)	14 (14.4)	14 (14.4)	49 (50.5)
Doctors (GPs)	5 (7.7)	10 (15.4)	11 (16.9)	10 (15.4)	29 (44.6)
Experts	0	2 (6.7)	2 (6.7)	12 (40.0)	14 (46.7)
***Total***	*54 (4.8)*	*88 (7.8)*	*139 (12.3)*	*180 (16.0)*	*665 (59.1)*

^**a**^body size identified as “obese” on the picture

### Perceptions of own weight

**Fig. 2:**
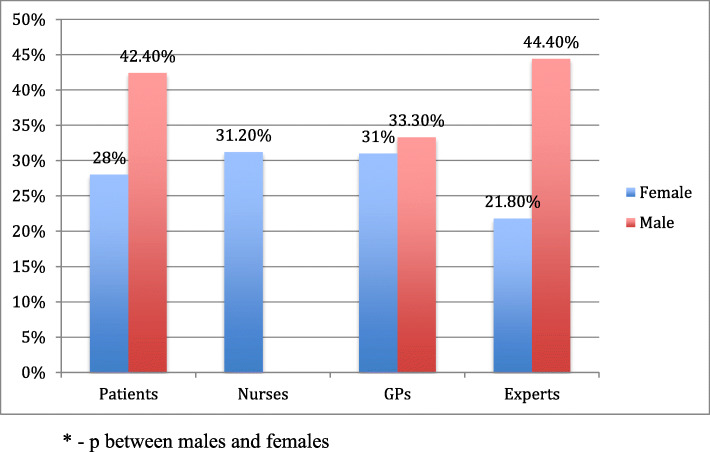
The rate of their own body weight misperception by gender and different groups of respondents

Most of the participants (69.9%) with BMI 25.0–29.9 correctly identified that they were overweight (Table 3). Overweight nurses and doctors were better to perceive their weight category as compared to patients and PHE of whom 21.2 and 27.3%, respectively, underestimated their weight. Nearly similar percentage (63.5%) of participants with obesity responded that they are very overweight, the rest stated that they have overweight (Table 3). There was no significant difference between the study groups. Males tend to have a higher rate of weight misperception as compared to females (Fig. 2). Only male doctors had similar weight misperception rate as female doctors. Among overweight respondents, significantly more have overestimated their figure BMI than underestimated, without larger difference between study groups. Majority of respondents with obesity in all groups have chosen correct figures (Table 4).

**Table 3:** Perception of own weight: distribution of answers of respondents who reported their own body image by BMI categories

Group of respondents by BMI categories^**a**^	Perception of own weight^**b**^, n (%)				Total
	Answer of respondent				
	I am Underweight	About the right weight	A bit overweight	Very overweight	
**BMI 18.5–24.9**					
Patients	37 (9.2)	281 (70.1)	82 (20.4)	1 (0.2)	*401 (100)*
Nurses	2 (3.8)	32 (60.4)	19 (35.8)	0	*53 (100)*
Doctors	1 (3.8)	17 (65.4)	8 (30.8)	0	*26 (100)*
Policy makers	0	11 (91.7)	1 (8.3)	0	*12 (100)*
*Total*	*40 (8.1)*	*341 (69.3)*	*110 (22.4)*	*1 (0.2)*	*492 (100)*
**BMI 25.0–29.9**					
Patients	0	63 (21.2)	202 (68.0)	32 (10.8)	*297 (100)*
Nurses	0	0	24 (96.0)	1 (4.0)	*25 (100)*
Doctors	0	2 (8.0)	18 (72.0)	5 (20.0)	*25 (100)*
Policy makers	0	3 (27.3)	5 (45.5)	3 (27.3)	*11 (100)*
*Total*	*0*	*68 (19.0)*	*249 (69.9)*	*41 (11.5)*	*358 (100)*
**BMI ≥ 30**					
Patients	0	5 (2.5)	67 (33.8)	126 (63.6)	198 (100)
Nurses	0	0	8 (42.1)	11 (57.9)	19 (100)
Doctors	0	0	4 (30.8)	9 (69.2)	13 (100)
Policy makers	0	0	1 (33.3)	2 (66.7)	3 (100)
*Total*	*0*	*5 (2.1)*	*80 (34.3)*	*148 (63.5)*	233 *(100)*

^a^BMI was calculated by self-reported weight and height

^b^Perception of own weight were asked by question: Which of the following best describes how you think of yourself at the moment?

**Table 4:** Perception of own weight: distribution of answers of respondents who identified own body image by the scale of pictures

Group of respondents^a^	BMI of the body images chosen^**b**^, n (%)			Total
	< 25	25–30	> 30	
**BMI 18.5–24.9**				
Patients	244 (60.8)	134 (33.4)	23 (5.7)	*401 (100)*
Nurses	29 (54.7)	22 (41.5)	2 (3.8)	*53 (100)*
Doctors	20 (76.9)	4 (15.4)	2 (7.7)	*26 (100)*
Policy makers	9 (75.0)	3 (25.0)	0	*12 (100)*
Total	302 (61.4)	163 (33.1)	27 (5.5)	*492 (100)*
**BMI 25.0–29.9**				
Patients	27 (9.1)	111 (37.5)	158 (53.4)	*296 (100)*
Nurses	1 (4.0)	14 (56.0)	10 (40.0)	*25 (100)*
Doctors	1 (4.0)	11 (44.0)	13 (52.0)	*25 (100)*
Policy makers	1 (9.1)	5 (45.5)	5 (45.5)	*11 (100)*
*Total*	*30 (8.4)*	*141 (39.5)*	*186 (52.1)*	*357 (100)*
**BMI ≥ 30**				
Patients	4 (2.0)	19 (9.6)	175 (88.4)	*198 (100)*
Nurses	0	5 (26.3)	14 (73.7)	*19 (100)*
Doctors	0	1 (7.7)	12 (92.3)	*13 (100)*
Policy makers	0	0	3 (100)	*3 (100)*
*Total*	*4 (1.7)*	*25 (10.7)*	*204 (87.6)*	*233 (100)*

^a^BMI was calculated by self-reported weight and height

^b^Category of body weight by the answers of respondents who identified own body image by the scale of pictures

### Satisfaction with the own body weight

**Fig. 3:**
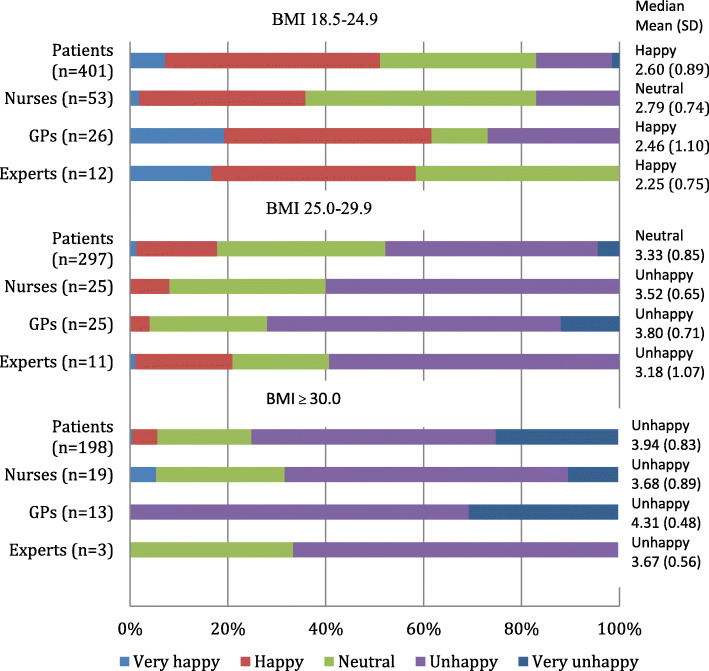
Satisfaction about current own body weight among respondents by BMI category in different groups of respondents

The feelings about current weight were significantly different between those who had normal BMI as compared to individuals with overweight/obesity. Participants with normal BMI were mostly happy while those who had overweight/obesity were mostly unhappy with their weight (Fig. 3). Among respondents with overweight/obesity doctors had the highest mean scores (3.80/4.31) showing that this group was more disappointed with their weight than other participant groups. The mean scores for patients, nurses and PHE with overweight/obesity were 3.33/3.94, 3.52/3.68 and 3.18/3.67, respectively.

### Understanding of causes and health risks associated with obesity

**Fig. 4:**
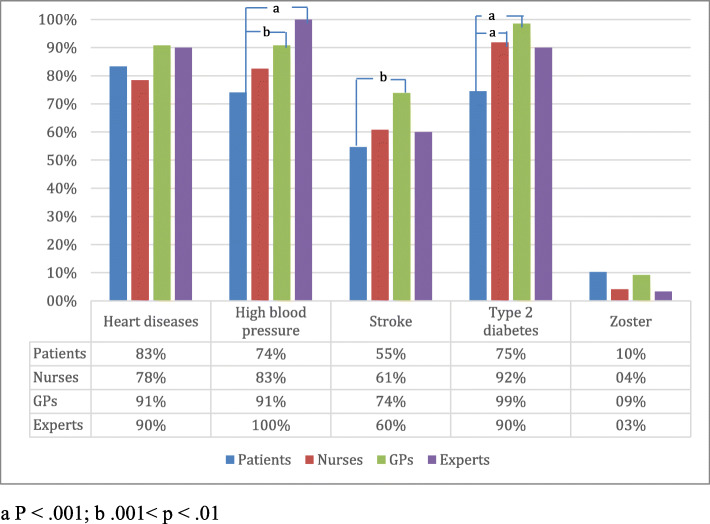
Awareness of respondents how chronic diseases are associated with obesity: percentage of respondents who reported the listed diseases

**Fig. 5:**
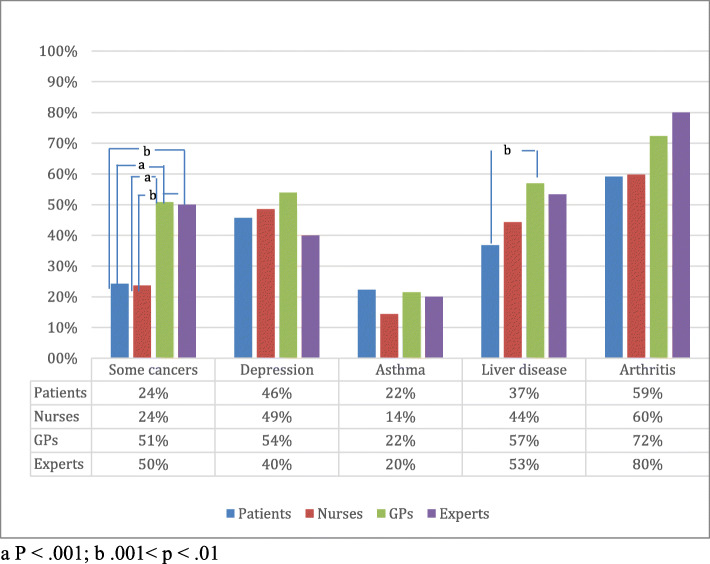
Other diseases associated with obesity (% say more likely to get, if obese)

Majority of respondents were aware that obesity is associated with increased risk of 5 health outcomes - heart diseases, high blood pressure and stroke, as well as T2DM and arthritis (Figs. 4 and 5). GPs showed the highest awareness of mentioned health conditions related to obesity (rate of correct answers 91, 91, 74, 99 and 72%, respectively). Patients also presented quite high percentage of correct answers – 83, 74, 55, 75 and 59%, respectively.

The respondents in all groups were less aware of other risk factors associated with obesity (Fig. 5). Less than half of respondents thought that obesity is a risk factor for depression. Half of PHE and GPs answered that obesity is associated with the increased risk of some cancers, while only quarter of patients and nurses identified this relationship. Was included to show for the respondents that not all presented diseases are associated with obesity. Majority in all groups have correctly answered the reference question that zoster is not associated with obesity.

**Fig. 6:**
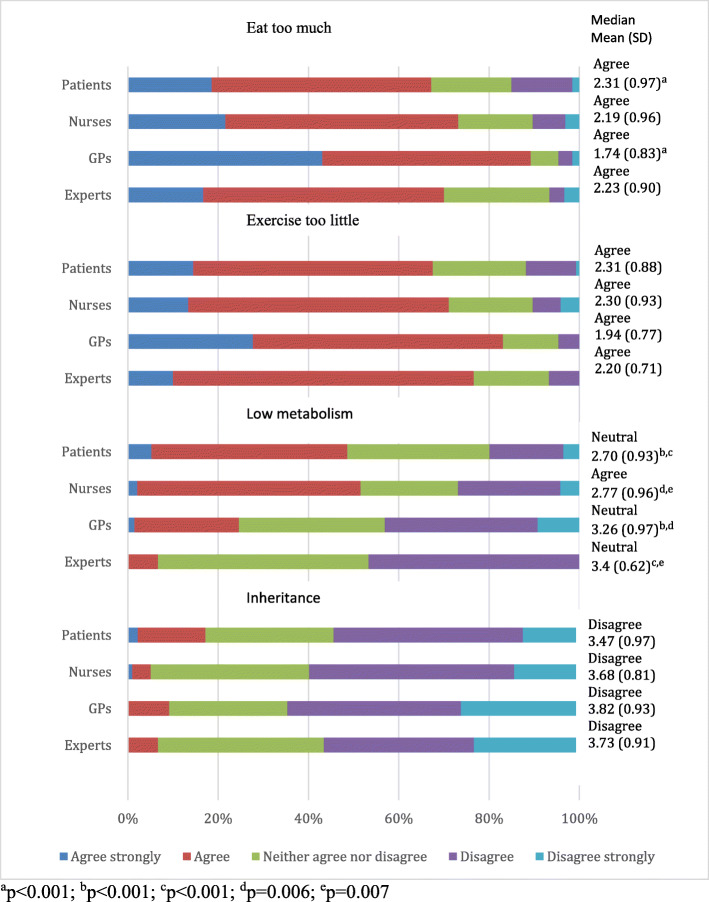
Beliefs about the causes of obesity by respondent groups

Most of the respondents in all groups agreed that eating too much and exercising too little causes obesity (Fig. 6). Nearly half of patients (48.6%) and nurses (51.6%) agreed that low metabolism is a cause of obesity. However, significantly less PHE (6.7%) and GPs (24.6%) agreed with such statement.

### Factors associated with lifestyle changes

About one third of respondents in all groups reported the changes in their lifestyle during last 12 months (Table 1). Majority of them started more healthier diet and increased physical activity.

Univariate and multivariate analysis have demonstrated that overweight individuals were more likely to change their lifestyle, if they were younger than 45 years (OR 1.64, 1.06–2.55; *p* = 0.025) and less likely, if they overestimated their current weight (OR 0.44, 0.20–0.96; *p* = 0.036) (Table 5). Individuals with obesity were engaged in lifestyle changing activities, if they were females (OR 1.77, 1.01–3.10; *p* = 0.047), had higher education (1.81, 1.05–3.10; *p* = 0.031) and were unhappy with their current weight (2.57, 1.36–4.84; *p* = 0.003). Individuals with obesity were less likely to make changes in their lifestyle, if they underestimated their current weight (0.49, 0.28–0.84; *p* = 0.010). Only unhappiness with the current weight was found to be statistically significant independent factor. Realistic perception of others and own body size, knowledge about diseases related to weight and causes of obesity as well as respondents’ group and incomes had no significant association with healthier behavior among individuals with overweight/obesity (Table 5).

**Table 5:** Odds ratios for lifestyle changes during last 12 months

Variable	Overweight			Obese		
	OR	95% CI	*p* value	OR	95% CI	*p* value
Respondents’ group (health care providers and public health experts)	0.68	0.37–1.23	0.198	1.10	0.54–2.27	0.790
Sex (female)	1.09	0.68–1.76	0.727	1.77	1.01–3.10	0.047
Age (< 45)	1.64	1.06–2.55	0.025^a^	0.81	0.47–1.39	0.435
Education (higher)	0.68	0.43–1.06	0.090	1.81	1.05–3.10	0.031
Income (> = 500)	0.99	0.64–1.54	0.963	0.84	0.50–1.41	0.514
Identified correct figure of obese individual:						
Female	1.22	0.63–2.36	0.560	1.03	0.49–2.17	0.937
Male	1.29	0.59–2.81	0.528	1.55	0.63–3.77	0.337
Perception of own weight						
Underestimated	0.73	0.41–1.29	0.279	0.49	0.28–0.84	0.010
Overestimated	0.44	0.20–0.96	0.036^a^	–	–	–
Perception of own figure						
Underestimated	1.20	0.53–2.70	0.669	1.53	0.70–3.38	0.286
Overestimated	1.00	0.63–1.57	0.981	–	–	–
Happiness with own weight (unhappy)	1.11	0.72–1.72	0.628	2.57	1.36–4.84	0.003^a^
Recognizing health risks associated with obesity (less than 2 cardiovascular and 2 other risk factors)	0.69	0.45–1.07	0.096	1.11	0.66–1.86	0.699
Causes of obesity:						
Inheritance (disagree/neutral-agree)	1.06	0.69–1.63	0.797	0.64	0.38–1.10	0.101
Low metabolism (disagree/neutral-agree)	0.81	0.49–1.34	0.408	0.91	0.44–1.86	0.790
Eats too much (disagree/neutral-agree)	0.89	0.54–1.47	0.639	1.12	0.65–1.94	0.675
Exercise too little (disagree/neutral-agree)	1.17	0.73–1.86	0.520	1.13	0.64–2.00	0.677

^a^Independent risk factors identified in multivariate analysis

## Discussion and conclusion

### Discussion

Our study showed that significant visual body size misperception exists in all respondent groups in estimating others weight, however, they were reasonably accurate in estimating their own body weight. Majority of respondents share similar attitudes about the causes of obesity, knows major cardiovascular diseases associated with obesity, however, the knowledge about other risks of obesity is inadequate. Age, realistic weight perception and feelings about own weight are associated with lifestyle changing behavior.

The first step in treating obesity is to diagnose it and clearly communicate diagnosis to the patient. Our study reveals that healthcare professionals and PHE have similar level of visual body size misperception as the patients. People tend to recognize visually only extreme, high-risk class III obesity and neglect early stages. One possible explanation could be that in general there is a greater acceptance of heavier body weights in the population [12]. Beside this, people make choices for food consumption based on how individual weight compares to social norms [13]. In the questionnaire that was used in the current study there was the question how people feel about their weight. None of the overweight nurses and only 8% doctors with overweight replied that they are “about the right weight”. In contrast, about one fifth of the overweight patients and one fourth of overweight PHE have chosen this answer. Similar results were established in the National Health and Nutrition Examination Survey (NHANES) in the United States. Twenty four percent of overweight individuals from US civilian population thought that their weight was about the right [14].

Only 2.5% of patients with obesity in our study replied that their weight is about the right, while nobody among healthcare providers or PHE have chosen this definition for their current weight. By contrast, 3% of women and 12% of males with obesity in US population thinks that they have about the right weight [15]. Participants with obesity in our study were less likely to be engaged in lifestyle change, if they underestimated their weight category. Underestimation of weight category maybe caused due to the lack of information about personal weight and obesity definition, or because of the belief that their weight is normal. However, the information about real weight status provided by physicians increases likelihood that the patients with overweight/obesity will attempt to lose weight [16].

Individual’s body size perception was evaluated asking which figure represents their body shape best. More than half of the overweight individuals overestimated their body size. Such findings correlate well with the data from the other studies [17, 18]. Overestimation of body size may have a negative influence on self-esteem and rise psychological, social and dietary issues [19]. It must be noted that female patients perceive their body size more realistically than males. Male patients think that they are thinner than they are, and this is more evident with increasing body size or weight status [20]. The other aspect is feelings about individual body size. Several studies have pointed on direct relation between increasing BMI and dissatisfaction with the body size [21, 22]. Moreover, these studies also revealed that dissatisfaction with body size is an important motivator for engaging in weight loss activities. However, feelings about height or specific body areas may also be included in the concept of satisfaction with body size [23]. In our study we narrowed the idea of body size and asked participants only how they feel about their current weight. Individuals with obesity were mostly unhappy with their weight and only 5% of patients and nurses responded that they are happy with their current weight. Results similar to those from current international study by Caterson ID et al. [24] where only 6% of patients with obesity were happy with their weight. Respondents with obesity, but not overweight, were more likely to change their lifestyle if they were dissatisfied with their current weight.

Several theories and conceptual models are used to understand health behavioral change [25]. In most of them motivation to change is based on attitudes, possible benefits, perceived threat or risk of a specific condition, or desire to achieve positive outcomes. Motivation alone without intentions is not enough to change lifestyle. GPs may form appropriate intentions however, it is important to consider how much intended behaviour is driven by attitudinal or normative considerations, or by feelings of perceived behavioral control [26]. Moreover, patients are more likely to be engaged in a particular behavior, if it is presented as an action with a target, performed within a given context and at a certain point in time [26]. If the patients already intend to act, it is unlikely that they need more information. However, if the patients are going to be engaged in the class of behaviors such as lifestyle change, they may need stronger motivation, proper context and much longer time. Awareness of the risks associated with obesity may motivate people to change lifestyle. The results of our study as well as the data from the literature reveal that individuals and health care providers recognizes heart disease, high blood pressure and T2DM as adverse health consequences of obesity [27–29]. However, nearly half of the PHE and GPs failed to recognize association of obesity with some types of cancer and depression, and even more with asthma. The knowledge of the nurses about obesity as a risk factor was similar to that of the patients. French study has found that GPs who subscribed medical journals or have taken CME courses about the management of weight problems felt more effective in treating individuals with obesity [29]. Failure to recognize diseases associated with obesity may be a barrier for weight loss counseling especially in overweight patients. Also, misperception of risks associated with obesity may prevent strong action on the society level.

In this study respondent beliefs about biological and lifestyle factors as a cause of obesity was investigated. All respondent groups agreed that exercising too little and eating too much causes obesity. Moreover, all groups disagreed that inheritance is a cause of obesity. Similar results were found in Caterson ID et al. study [24] where patients and healthcare providers emphasized life-style related factors, but not a genetic predisposition as a barrier to weight loss. By contrast, it is now widely recognized that obesity has a genetic predisposition and obesogenic environment increases genetic risk for obesity [30]. In general, influence of genes has to be discussed with the patient as this may diminish self-blame and the patients have to be informed that genetic risk for obesity maybe reduced by increasing physical activity and avoiding some specific dietary components [24, 30].

One third of individuals with overweight and half of those with obesity have changed their lifestyle during last 12 month in our study population and there was no difference between patients, healthcare providers and PHE. In comparison, 61% of US adults with obesity over the last 12 months tried to lose weight [31]. Among overweight individuals those who were younger than 45 years were more likely to change their lifestyle. Individuals with obesity were more prone to adopt healthier lifestyle if they had higher education, were females and were unhappy with their weight. Beside higher education and female gender, increasing BMI, insurance coverage, comorbidities such as diabetes or arthritis and Hispanic race were associated with more weight loss activities among US adults with obesity [31]. Overestimation of weight among individuals with overweight and underestimation among those with obesity precluded lifestyle changes in our study population. These data are similar to Duncan DT et al. [32] findings that weight misperception was a strong predictor of weight loss activities for both genders and all racial/ethnic groups among US adults with overweight and obesity.

This study explored attitudes about obesity in different social groups. Healthcare providers as well as PHE in general have similar attitudes to those of general population. It seems that attitudes and beliefs rooted in society is hard to change even with specialized education. More focused teaching should be aimed at paradigm shift. The people fail to recognize obesity in the others in an early stage, when weight control can be more efficient. People themselves tend to be realistic about their weight. The data from this study and the literature show, that individuals with overweight and obesity are more motivated to lose weight, if they have realistic perception of their weight. But they need support. Recent international multicenter study found that it took a median of 3 years and a mean of 6 years between the time when individuals started struggling with excess weight or obesity and when they had a first weight management conversation with a healthcare provider. Moreover, 46% of individuals have initiated discussion themselves [24]. BMI estimation and assessment of individuals’ health profile maybe an effective way to start such conversations [33]. Timely interventions may prevent potential complications of obesity. Furthermore, education may help for individuals to understand less apparent relationship between obesity and health. The lack of information is likely to compromise patients’ abilities to make informed choices about their health. Swift JA et al. [34] observed that intended weight loss was positively associated with health beliefs. However, in our study knowledge about obesity as a risk factor for cardiovascular and non-cardiovascular diseases had no impact on healthier behavior.

Our study has some limitations. First, the majority of the patient respondents questioned were women. This could be due to the fact that in overall, percentage of women in the population is higher (especially in age over 60), also women are covered by larger number of prevention programs and attend GPs office more frequently. In addition, they do accept invitation to participate in the survey more positively in comparison with men. Second, the primary care centers that participated in our survey are teaching centers and are affiliated with Lithuanian University of Health Sciences. In all centers family medicine residents have their practice. Thus, GPs and nurses who participate in the teaching activities may have more knowledge than healthcare providers from other primary care offices. Third, we used self-reported weight and height to estimate BMI of the patients, and this approach is subject to the bias [35]. However, the percentage of individuals with overweight and obesity among the respondents was similar to the data from population studies performed in Lithuania [36]. Fourth, we did not ask participants, if adoption of healthier lifestyle was motivated by necessity to lose weight. By presenting a broader question we intended to cover also individuals whose primary goal was to increase physical activity and/or adopt healthier diet not only for weight loss purposes. Finally, from cross-sectional survey data we were unable to establish causal association between body weight satisfaction and healthier lifestyle. It is unclear whether dissatisfaction with weight motivates to change lifestyle or change in lifestyle alters perception of weight and results in the feelings of dissatisfaction.

### Conclusion

Participants from different groups have similar body size perception and knowledge about obesity. Younger age and overestimated current weight are associated with healthier behavior among overweight individuals and disappointment with current weight among participants with obesity. Diagnosing obesity only by visual means is inaccurate. BMI estimation during the visit to primary care office is simple and important tool that facilitates diagnosis of obesity. Combining information from perceived body image with weight status and feelings about current weight may give more insight into individual’s views and motivation to adopt healthier lifestyle behavior. Serious knowledge gaps exist among healthcare providers about the impact of obesity on non-cardiovascular diseases. In the age when the amount of information in medicine grows exponentially, continuous education of healthcare providers is vital.

## Data Availability

Data could be provided by the request to authors of manuscript.

## Abbreviations

BMI: Body mass index
NHANES: National Health and Nutrition Examination Survey
US: United States
T2DM: Type 2 diabetes mellitus
PHE: Public health experts
GPs: General practitioners
CME: Continuing medical education

## References

[1] WHO (2019). Obesity and overweight.

[2] Fontaine KR, Redden DT, Wang C, Westfall AO, Allison DB (2003). Years of life lost due to obesity. JAMA.

[3] Finkelstein EA, Fiebelkorn IC, Wang G. National medical spending attributable to overweight and obesity: how much, and who's paying? Health Aff. 2003; Suppl1: Web Exclusives, W3-219-26. 10.1377/hlthaff.w3.219 14527256

[4] Ritten A, LaManna J (2017). Unmet needs in obesity management: from guidelines to clinic. J Am Assoc Nurse Pract.

[5] Ma J, Xiao L, Stafford RS (2009). Adult obesity and office-based quality of care in the United States. Obesity (Silver Spring).

[6] Bleich SN, Pickett-Blakely O, Cooper LA (2011). Physician practice patterns of obesity diagnosis and weight-related counseling. Patient Educ Couns.

[7] Bertakis KD, Azari R (2005). The impact of obesity on primary care visits. Obes Res.

[8] Volpp KG, Mohta NS. Patient engagement survey: the failure of obesity efforts and the collective nature of solutions. NEJM Catal. 2018; https://catalyst.nejm.org/doi/full/10.1056/CAT.18.0092.

[9] Johnson F, Cooke L, Croker H, Wardle J (2008). Changing perceptions of weight in Great Britain: comparison of two population surveys. BMJ.

[10] Oldham M, Robinson E (2016). Visual weight status misperceptions of men: why overweight can look like a healthy weight. J Health Psychol.

[11] Curtice J. Attitudes to obesity: findings from the 2015 British social attitudes survey. Nat Cen Soc Res. 2015.

[12] Robinson E, Christiansen P (2014). The changing face of obesity: exposure to and acceptance of obesity. Obesity (Silver Spring).

[13] Burke MA, Heiland F (2007). Social dynamics of obesity. Econ Inq.

[14] Han L, You D, Zeng F, Feng X, Astell-Burt T, Duan S (2019). Trends in self-perceived weight status, weight loss attempts, and weight loss strategies among adults in the United States, 1999-2016. JAMA Netw Open.

[15] Burke MA, Heiland FW, Nadler CM (2010). From “overweight” to “about right”: evidence of a generational shift in body weight norms. Obesity (Silver Spring).

[16] Post RE, Mainous AG, Gregorie SH, Knoll ME, Diaz VA, Saxena SK (2011). The influence of physician acknowledgment of patients’ weight status on patient perceptions of overweight and obesity in the United States. Arch Intern Med.

[17] Paul T, Sciacca R, Bier M, Rodriguez J, Song S, Giardina E-G (2015). Size misperception among overweight and obese families. J Gen Intern Med.

[18] Lerner HM, Klapes B, Mummert A, Cha E (2016). Eat Weight Disord.

[19] Jauregui-Lobera I, Ezquerra-Cabrera M, Carbonero-Carreno R, Ruiz-Prieto I (2013). Weight misperception, self-reported physical fitness, dieting and some psychological variables as risk factors for eating disorders. Nutrients.

[20] Pulvers KM, Kaur H, Nollen NL, Greiner KA, Befort CA, Hall S (2008). Comparison of body perceptions between obese primary care patients and physicians: implications for practice. Patient Educ Couns.

[21] Anderson LA, Eyler AA, Galuska DA, Brown DR, Brownson RC (2002). Relationship of satisfaction with body size and trying to lose weight in a national survey of overweight and obese women aged 40 and older, United States. Prev Med.

[22] Millstein RA, Carlson SA, Fulton JE, Galuska DA, Zhang J, Blanck HM (2008). Relationships between body size satisfaction and weight control practices among US adults. Medscape J Med.

[23] Grogan S (2006). Body image and health: contemporary perspectives. J Health Psychol.

[24] Caterson ID, Alfadda AA, Auerbach P, Coutinho W, Cuevas A, Dicker D (2019). Gaps to bridge: misalignment between perception, reality and actions in obesity. Diabetes Obes Metab.

[25] Baranowski T, Cullen KW, Nicklas T, Thompson D, Baranowski J (2003). Are current health behavioral change models helpful in guiding prevention of weight gain efforts?. Obes Res.

[26] Fishbein M (2008). A reasoned action approach to health promotion. Med Decis Mak.

[27] Lake Snell Perry & Associates (2003). Obesity as a public health issue: a look at solutions: results from a national poll.

[28] Soriano R, Ponce de León Rosales S, García R, García-García E, Méndez JP (2012). High knowledge about obesity and its health risks, with the exception of cancer, among Mexican individuals. J Cancer Educ.

[29] Bocquier A, Verger P, Basdevant A, Andreotti G, Baretge J, Villani P (2005). Overweight and obesity: knowledge, attitudes, and practices of general practitioners in France. Obes Res.

[30] Goodarzi MO (2018). Genetics of obesity: what genetic association studies have taught us about the biology of obesity and its complications. Lancet Diabetes Endocrinol.

[31] Stokes A, Collins JM, Grant BF, Hsiao CW, Johnston SS, Ammann EM (2018). Prevalence and determinants of engagement with obesity care in the United States. Obesity (Silver Spring).

[32] Duncan DT, Wolin KY, Scharoun-Lee M, Ding EL, Warner ET, Bennett GG (2011). Does perception equal reality? Weight misperception in relation to weight-related attitudes and behaviors among overweight and obese US adults. Int J Behav Nutr Phys Act.

[33] Speer SA, McPhillips R (2018). Initiating discussions about weight in a non-weight-specific setting: what can we learn about the interactional consequences of different communication practices from an examination of clinical consultations?. Br J Health Psychol.

[34] Swift JA, Glazebrook C, Anness A, Goddard R (2009). Obesity-related knowledge and beliefs in obese adults attending a specialist weight-management service: implications for weight loss over 1 year. Patient Educ Couns.

[35] Connor Gorber S, Tremblay M, Moher D (2007). Gorber B. a comparison of direct vs. self-report measures for assessing height, weight and body mass index: a systematic review. Obes Rev.

[36] Grabauskas V, Klumbienė J, Petkevičienė J, Šakytė E, Kriaučionienė V, Veryga A (2015). Health behaviour among Lithuanian adult population, 2014.

